# Advanced Glycation End Products Induce Caudal Disc Degeneration in Ovariectomized Female Rats

**DOI:** 10.1002/jsp2.70114

**Published:** 2025-09-09

**Authors:** Xiao Liang, Zhaohui Li, Pengcheng Ren, Ze Gao, Xiaoming Tian, Wei Zhang, Justin Cooper‐White, Guobin Liu, Sidong Yang

**Affiliations:** ^1^ Department of Emergency Hebei Medical University Third Hospital Shijiazhuang People's Republic of China; ^2^ Department of Orthopaedic Surgery Hebei General Hospital Shijiazhuang People's Republic of China; ^3^ Department of Joint Surgery Peking University Ninth School of Clinical Medicine, Beijing Shijitan Hospital, Capital Medical University Beijing People's Republic of China; ^4^ Department of Spine Surgery Hebei Medical University Third Hospital Shijiazhuang People's Republic of China; ^5^ Department of Spine Surgery Tianjin Union Medical Center, The First Affiliated Hospital of Nankai University Tianjin People's Republic of China; ^6^ School of Chemical Engineering The University of Queensland Brisbane Queensland Australia; ^7^ Department of Orthopaedic Surgery The First Hospital of Hebei Medical University Shijiazhuang People's Republic of China; ^8^ Department of Orthopaedic Surgery Hebei Medical University Third Hospital Shijiazhuang People's Republic of China; ^9^ Hebei International Joint Research Centre for Spinal Diseases Shijiazhuang People's Republic of China

**Keywords:** estrogen, intervertebral disc degeneration, rat advanced glycation end products

## Abstract

**Background:**

Preclinical animal models are indispensable for the development of new therapeutic strategies and the study of the pathological mechanisms of intervertebral disc (IVD) degeneration (IVDD). This study aims to develop a reliable and reproducible rat model of IVDD by injecting advanced glycation end products (AGEs) into the IVD of ovariectomized rats.

**Methods:**

Twenty‐eight female Sprague–Dawley rats were allocated into the 31G needle group, vehicle group, 0.5 μg AGEs group, 1 μg AGEs group, 2 μg AGEs group, 4 μg AGEs group, and non‐ovariectomy group (*n* = 4). The coccygeal discs of the 31G needle group were punctured only, while the coccygeal discs of the vehicle group were injected with 1 μL PBS. The coccygeal discs of the AGEs groups underwent injection of AGEs at 0.5, 1, 2, and 4 μg, respectively. The coccygeal discs of the non‐ovariectomy group were injected with 2 μg AGEs. Rats in all groups, except for the non‐ovariectomy group, underwent bilateral ovariectomy. Two weeks later, the rat caudal models were evaluated using radiological examination, histological staining, and immunohistochemistry (IHC).

**Results:**

No signs of IVDD were found by radiological imaging, histology, or IHC in the 31G needle group or the vehicle group. By contrast, in the 0.5, 1, 2, and 4 μg AGEs groups, caudal IVDD was successfully established and the IVDD severity is increasing in a dose‐dependent manner. Compared with the 2 μg AGEs group, rats in the non‐ovariectomized group showed less IVDD, indicating the protective effect of endogenous estrogen on degenerative IVD.

**Conclusions:**

A single injection of AGEs to caudal discs can cause reliable and reproducible IVDD in ovariectomized female rats. Additionally, the endogenous estrogen might have a protective effect on the IVD to mitigate the degeneration.

## Graphical abstract

In this study, we established a reliable and reproducible rat model of IVD degeneration by injecting advanced glycation end products (AGEs) into the IVD of ovariectomized rats. We believe this easily handled preclinical animal model can not only benefit the studies on menopausal IVD degeneration but also oxidative damage‐induced IVD degeneration.

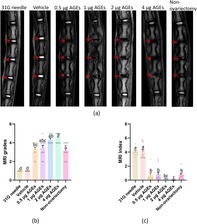

## 1 Introduction

Low back pain (LBP) is the leading cause of disability worldwide, with its prevalence escalating due to sedentary lifestyles and population aging. In 2020, it affected 619 million individuals globally, ranking as the top cause of years lived with disability [1]. Among the various causes of LBP, intervertebral disc (IVD) degeneration (IVDD) is a predominant factor [2].

The process of IVDD is characterized by a decrease of nucleus pulposus (NP) cells due to a variety of stimuli, resulting in an imbalance between the synthesis and degradation of extracellular matrix (ECM) [3]. The decrease in collagen type 2 (COL2) synthesis in the NP, the transition of the tissue composition to fibrosis, and the increase in matrix‐degrading enzymes all contribute to the loss of elasticity and mechanical integrity in IVD. Inflammation may also play a role [4, 5, 6, 7, 8]. So far, the development of new therapeutic strategies and the investigation of the pathological mechanism of IVDD have been slow. Preclinical animal models with good reproducibility would help to facilitate these studies. Current models include damage‐induced (e.g., needle puncture), mechanical stress, and spontaneous models (e.g., gene knockout) [9, 10, 11, 12, 13, 14]. These animal modeling protocols suffer from long modeling cycles, high costs, and complexity. Furthermore, the presence or absence rather than the severity of IVDD is often used as the endpoint in the studies of modeling protocols. There are no ideal animal modeling protocols used to produce a controlled degree of IVDD.

Advanced glycation end products (AGEs), derived from non‐enzymatic reactions between sugars and proteins, accumulate in processed foods and tissues, particularly under diabetic conditions [15, 16, 17]. AGEs are implicated in IVDD through oxidative stress and ECM degradation [18, 19, 20]. It has been documented that the accumulation of AGEs may affect the production of aggrecan and collagen in the NP and AF, leading to IVDD [21, 22]. Previous studies using AGEs in animal models required prolonged and repeated injections [23, 24].

It was found that estrogen has a protective effect on degenerated IVD [25, 26]. Ovariectomized female rats are often used to investigate the effects of estrogen on IVDD [10, 27]. The current rat IVDD models induced by AGEs are not well established, and the estrogenic effect on the IVD in this model is still unclear. In this study, we thus developed a caudal IVDD model using a single intradiscal injection of AGEs in ovariectomized female rats, aiming to validate the modified IVDD modeling protocol and the protective role of endogenous estrogens in the degenerative process of IVD.

## 2 Materials and Methods

### 2.1 Ethics

This study was approved by our ethics committee (Approval No. Z2022‐022‐1).

### 2.2 Animals and Experimental Design

A total of 28 healthy female Sprague–Dawley rats (11 weeks old, weighing 210–240 g and fed *ad libitum* with commercial pellet food and water) were divided into 7 groups (*n* = 4), including 31G needle, vehicle, 0.5, 1, 2, and 4 μg AGEs, and non‐ovariectomy groups (Table 1). Three coccygeal discs, Co7/8, Co8/9, and Co9/10, were used for injection surgery using a 10‐μL (31G needle, minimum scale 0.5 μL) microinjector (Shanghai Bolige Industry & Trade Co. China). All rats and commercial pellet feed were purchased from the Beijing HFK Bioscience Company Limited. Rats were subjected to surgical intervention 1 week after admission to the laboratory animal center (12 weeks of age).

**TABLE 1:** Grouping, coccygeal disc injection, and surgical protocols in rats.

Groups	Surgery	Dosage	Bilateral ovariectomy?
31G needle group	Puncture only	N	Y
Vehicle group	Injection of 1 μL PBS	1 μL	Y
0.5 μg AGEs group	Injection of 0.5 μg AGEs	1 μL	Y
1 μg AGEs group	Injection of 1 μg AGEs	1 μL	Y
2 μg AGEs group	Injection of 2 μg AGEs	1 μL	Y
4 μg AGEs group	Injection of 4 μg AGEs	1 μL	Y
Non‐ovariectomy group	Injection of 2 μg AGEs	1 μL	N

### 2.3 Ovariectomy

The rats were anesthetized by applying a 2% pentobarbital sodium intraperitoneal injection (the injection dose was 40 mg/kg). After successful anesthesia, the rats were placed in a lateral recumbent position, and the surgical site was sterilized with iodophor. A 1‐cm oblique incision was made to enter the abdominal cavity in the lateral abdomen, approximately 1 cm from the lower edge of the ribs and 1.5 cm from the anterior edge of the lumbar vertebrae. The cauliflower ovaries and fallopian tubes were first explored and identified. Next, the fallopian tubes were ligated, and the ovarian tissues were removed. Lastly, the surgical site was sutured layer by layer. Bilateral ovariectomy was performed in the same way.

### 2.4 Coccygeal IVD Injection

After completion of bilateral ovariectomy, the rats were immediately placed in a prone position. The palpation method was used to locate the coccygeal IVDs. The coccygeal IVD, Co5/6, was palpated over the skin where it was bordered by the fur part of the rat's tail. This was the initial location point. The second IVD, Co7/8, was palpated further distally. Mark Co7/8, Co8/9, and Co9/10 with a marker pen. The tail was sterilized with iodophor, and the dorsal skin of the corresponding site was incised. For the 31G needle group, the disc was punctured 4 mm deep using a microinjector with a homemade stopper and the needle was held for 10 s. For those groups with injections, rats were injected with the drug at the respective dosage (Table 1). During the injection surgery, the thumb and forefinger held both sides of the target disc so that the puncture site could be further defined, and the needle needed to enter perpendicular to the rat's tail to avoid damage to the endplate. The needle was held in the disc for 10 s after the drug injection to ensure that the drug entered the disc. Finally, the rat tail skin was sutured. All rats returned to their corresponding cages upon completion of surgery.

### 2.5 X‐Ray Examination

**FIGURE 1:**
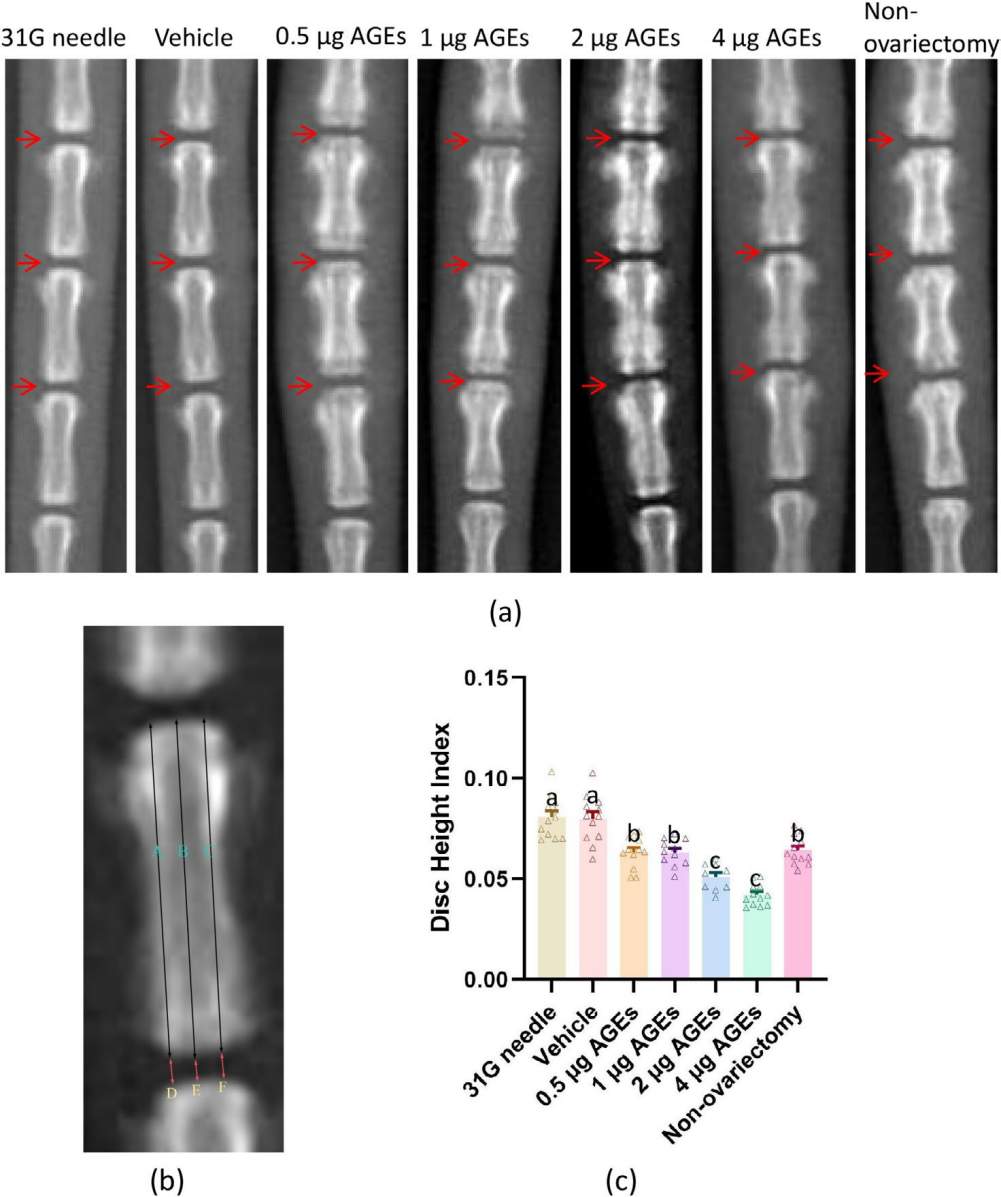
X‐ray images of rat coccygeal spine. (a) Schematic diagram of DHI measurement method: DHI = (D + E + F)/(A + B + C). (b) Representative X‐ray images of rat coccygeal spine (red arrows indicating targeted discs). (c) Statistical results of DHI in the coccygeal spine of rats in each group. Those labeled with the same letter indicate no statistical difference between groups, and those with different letters indicate a statistical difference between groups (*p* < 0.05).

All rats were subjected to coccygeal x‐ray coronal plane radiography 2 weeks after injection. Anesthetized rats were photographed with a Multitom RAX digital radiographic fluoroscopy system (voltage = 50 kV, current = 1.6 mAs) (Siemens Healthcare GmbH, Germany). ImageJ software (USA, 1.54f) was applied for the measurement of rat‐tail parameters. The measurements were all obtained by a similar x‐ray protocol reported by Hoogendoorn et al. [28], that is, the height of the IVD and proximal adjacent vertebrae was measured three times by using the left, center, and right centerline method. The average height of the IVD was divided by the average height of the proximal adjacent vertebrae to obtain the disc height index (DHI) (Figure 1a).

### 2.6 MRI Examination

All rats underwent coccygeal magnetic resonance imaging (MRI) sagittal videography after X‐ray examination. Anesthetized rats were placed prone in a 3.0 Tesla Clinical Magnet (PhlipsMedical Systems Nederland B.V., The Netherlands) with the tail in the center of the coil to obtain T2‐weighted images (repetition time = 3000 ms, echo time = 80 ms, slice thickness = 1 mm). The images were evaluated according to Pfirrmann classification [29]. Quantitative analysis of the area and signal intensity (gray values) of the NP was performed according to the protocol by Sobajima et al. [30]. Briefly, midsagittal images were analyzed using ImageJ software, and the NPs were outlined to define the region of interest (ROI). The MRI index was calculated based on the ROI area multiplied by the average signal intensity (gray value).

### 2.7 Histology

All rats were sacrificed by anesthetic overdose after completion of MRI scanning and X‐ray examination. The coccygeal spine column consisting of the three target IVDs was dissected and immersed in 4% paraformaldehyde for 48 h, followed by decalcification in 10% Ethylene Diamine Tetraacetic Acid (EDTA) for 60 days, with the EDTA solution refreshed every 2 days. The target discs were then made into paraffin blocks and sectioned in the sagittal plane using a microtome (Leica, Germany) to a thickness of 5 μm.

The paraffin sections were deparaffinized, rehydrated, and stained with hematoxylin and eosin (H&E) (G1120, Solarbio, China), Alcian blue (AB) (G1560, Solarbio, China), and Safranin O‐Fast green (SO/FG) (G1371, Solarbio, China). The histological staining was performed according to the manufacturer's instructions.

Using the histological staining images, histological scoring was performed according to a previously reported standardized histological grading scale [31], which was used to assess rats' IVDD degrees in terms of five aspects, including NP morphology, NP cellularity, AF morphology, AF/NP border, and endplates.

### 2.8 Immunohistochemistry

Paraffin sections were treated with trypsin for 1 h at 37°C. Peroxidase activity was blocked with hydrogen peroxide. Sections were incubated with rabbit monoclonal anti‐Aggrecan antibody (1:200, #DF7561, Affinity) and rabbit monoclonal anti‐SOD2 antibody (1:1000, 24 127‐1‐AP, Proteintech), respectively, at 4°C overnight. They were incubated with a reaction‐enhancing solution for 20 min, washed, and then incubated with an enhanced enzyme‐labeled goat anti‐rabbit IgG polymer for 20 min. Sections were incubated with diaminobenzidine for color development reactions. Finally, nuclei were stained with hematoxylin and differentiated for 15 s. The slides were treated with gradient alcohol and xylene and sealed with neutral resin. All images were taken under a light microscope. ImageJ software was applied to quantify and analyze the percentage of positive cells in SOD2 sections and the average optical density (AOD) of Aggrecan sections.

### 2.9 Statistical Analysis

Data analysis was conducted using the statistical software SPSS 26.0 (SPSS Inc., Chicago, IL, USA). DHI, AOD of aggrecan, and positive cell rate for SOD2 were determined by one‐way analysis of variance (ANOVA), followed by between‐group comparison using Tukey's multiple comparisons test. Pfirrmann's classification, MRI index, and histological grading scale were analyzed using Kruskal‐Wallis tests and compared between groups using Dunn's test. *p* < 0.05 was regarded as statistically significant.

## 3 Results

### 3.1 Rats

Of the 28 rats, one from the 2 μg AGEs group died post‐surgery due to anemia. In the 1 μg AGEs group, one IVD was excluded due to hemorrhage. Final sample sizes were 12 IVDs/group for most groups, except for 1 μg (11 IVDs) and 2 μg (9 IVDs).

### 3.2 X‐Ray Evaluation

By using ANOVA and Tukey's multiple comparisons test, we obtained comparative results of DHI in each group. No significant differences in DHI were observed between the vehicle group and 31G needle group. The AGEs groups showed significant DHI reductions compared to these controls, with higher AGEs doses correlating with greater reductions. The non‐ovariectomy group exhibited similar DHI decreases as the 0.5 μg group and 1 μg AGEs group (Figure 1b,c) (Table S1).

### 3.3 MRI Evaluation

**FIGURE 2:**
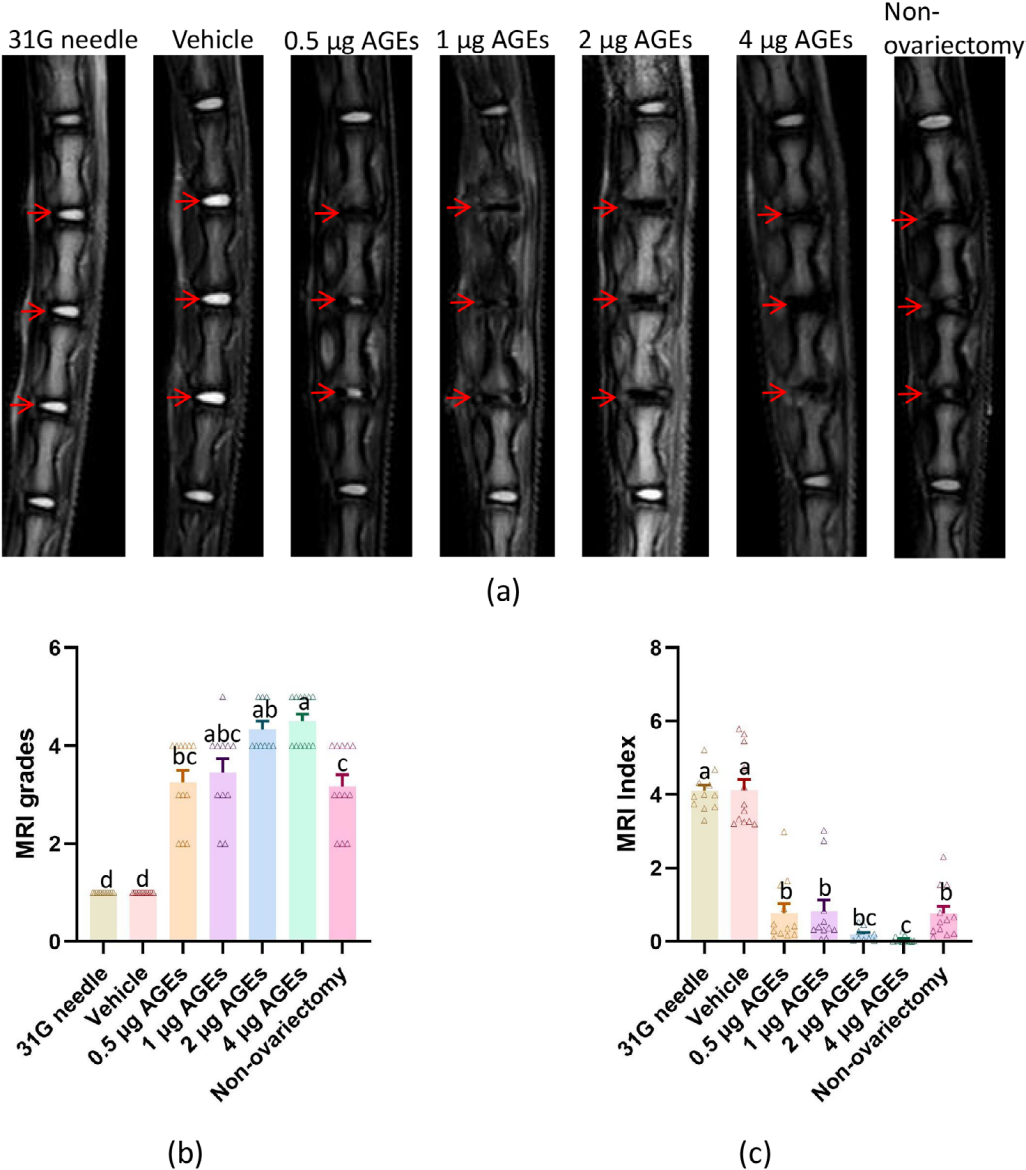
MRI scans of rat coccygeal spine. (a) Representative MRI images of rat coccygeal spine (red arrows indicating targeted discs). (b) Statistical results of Pfirrmann's grading in the coccygeal spine of rats in each group. (c) Statistical results of MRI index in the coccygeal spine of rats in each group. Those labeled with the same letter indicate no statistical difference between groups, and those with different letters indicate a statistical difference between groups (*p* < 0.05).

By Kruskal‐Wallis test and Dunn's test, we obtained the results of MRI comparison among the groups. MRI scans revealed no IVDD in the vehicle group or 31G needle group (Pfirrmann Grade 1). In contrast, the AGEs groups showed marked IVDD with increased Pfirrmann grades and decreased NP gray values. Higher AGEs doses corresponded to more severe IVDD (Figure 2) (Tables S2, S3).

### 3.4 Histological Evaluation

**FIGURE 3:**
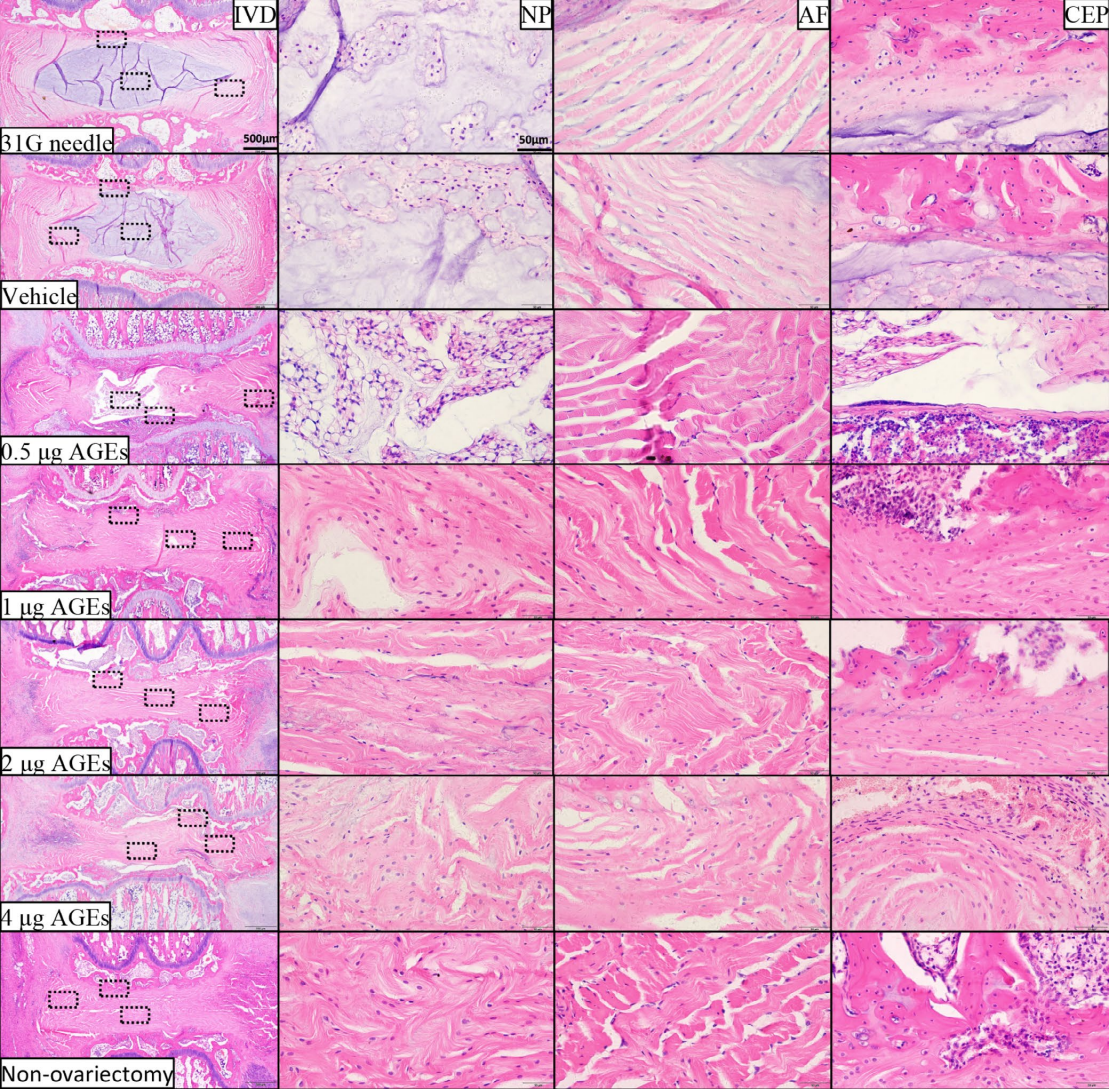
H&E staining of rat IVDs. From top to bottom: 31G needle group, vehicle group, 0.5 μg AGEs group, 1 μg AGEs group, 2 μg AGEs group, 4 μg AGEs group, and non‐ovariectomy group. AF, annulus fibrosus; CEP, cartilage endplate; H&E, hematoxylin and eosin; IVD, intervertebral disc; NP, nucleus pulposus.

**FIGURE 4:**
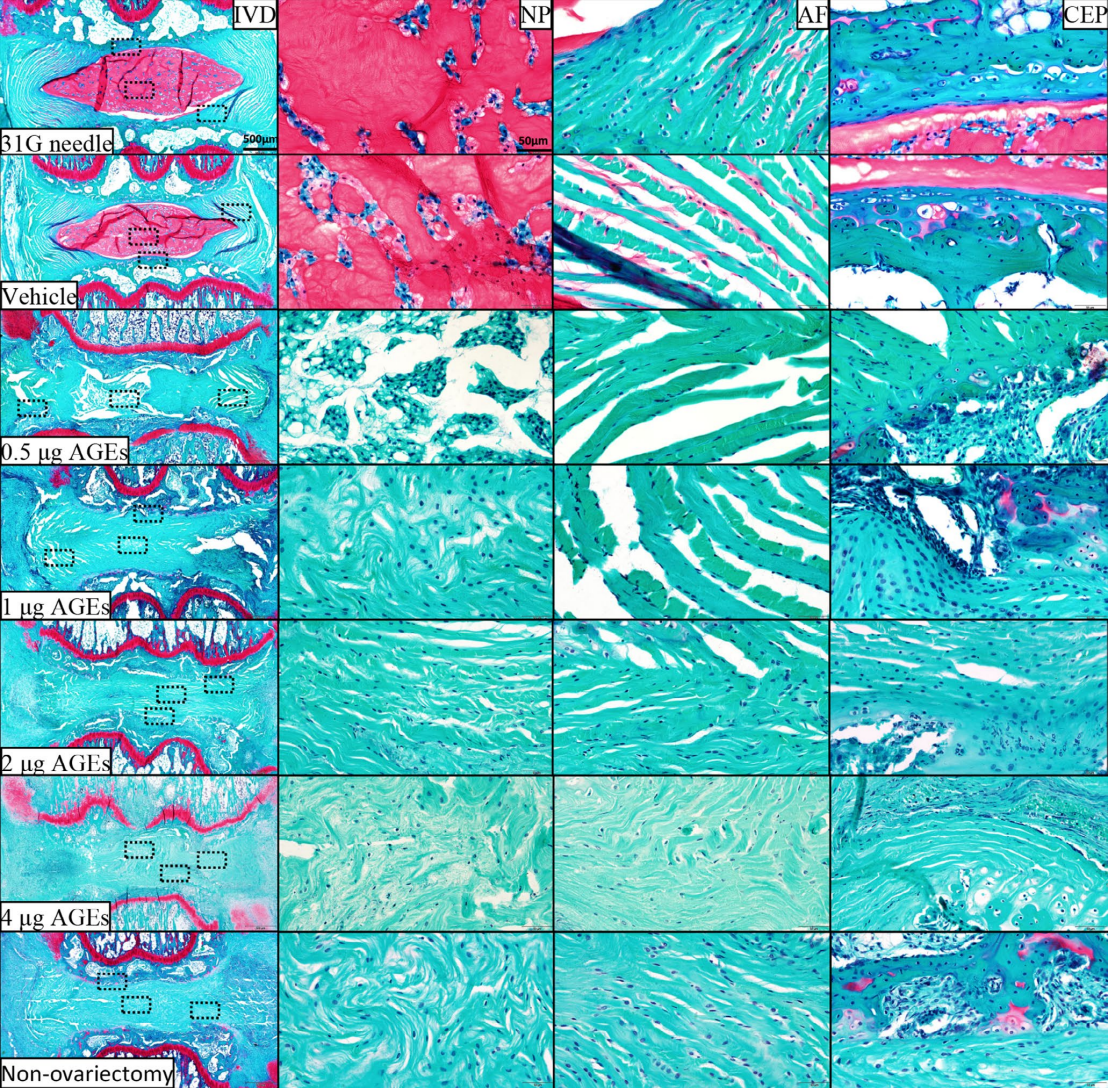
Safranin O‐Fast green staining of rat IVDs. From top to bottom: 31G needle group, vehicle group, 0.5 μg AGEs group, 1 μg AGEs group, 2 μg AGEs group, 4 μg AGEs group, and non‐ovariectomy group. AF, annulus fibrosus; CEP, cartilage endplate; IVD, intervertebral disc; NP, nucleus pulposus.

**FIGURE 5:**
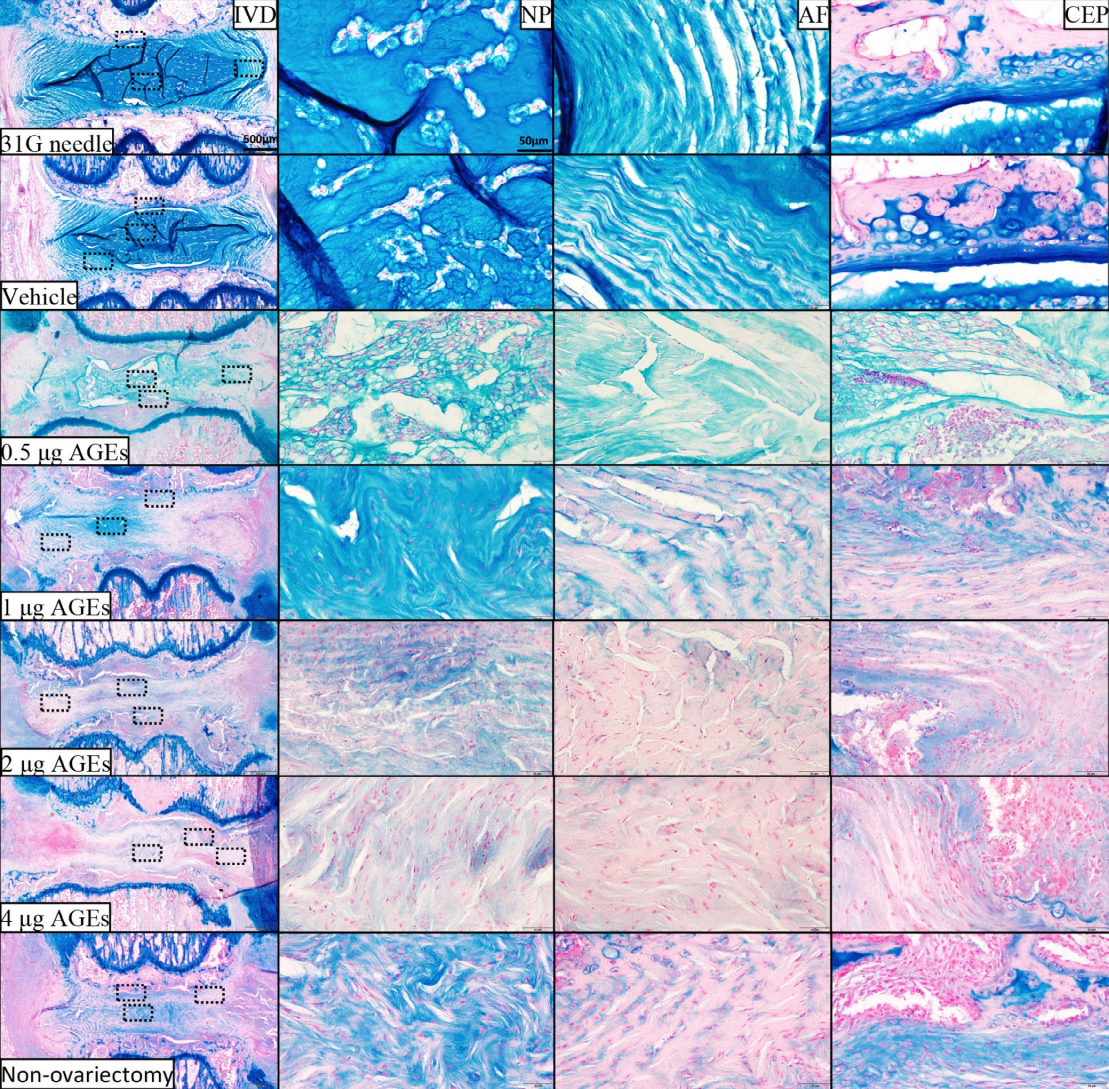
Alcian blue staining of rat IVDs. From top to bottom: 31G needle group, vehicle group, 0.5 μg AGEs group, 1 μg AGEs group, 2 μg AGEs group, 4 μg AGEs group, and non‐ovariectomy group. AF, annulus fibrosus; CEP, cartilage endplate; IVD, intervertebral disc; NP, nucleus pulposus.

By Kruskal‐Wallis test and Dunn's test, we obtained the results of histologic grading scale comparison among the groups. H&E (Figure 3), SO/FG (Figure 4), and AB staining (Figure 5) did not indicate IVDD in the vehicle group or 31G needle group. By contrast, in the 0.5 μg AGEs group, the coccygeal IVDs showed apparent signs of degeneration, including (1) the NP became irregular and reduced in extent; (2) the boundary between the NP and AF became blurred; and (3) the fibers in AF were slightly interrupted. In the 1 μg AGEs group and non‐ovariectomy group, disc degeneration was more obvious, with the disappearance of vacuolated NP cells and the NP/AF boundary. The 2 μg AGEs group and 4 μg AGEs group had severer disc degeneration—a large number of broken or serpentine‐patterned fibers appeared in the AF, and the endplates showed significant osteophytes or ossification.

**FIGURE 6:**
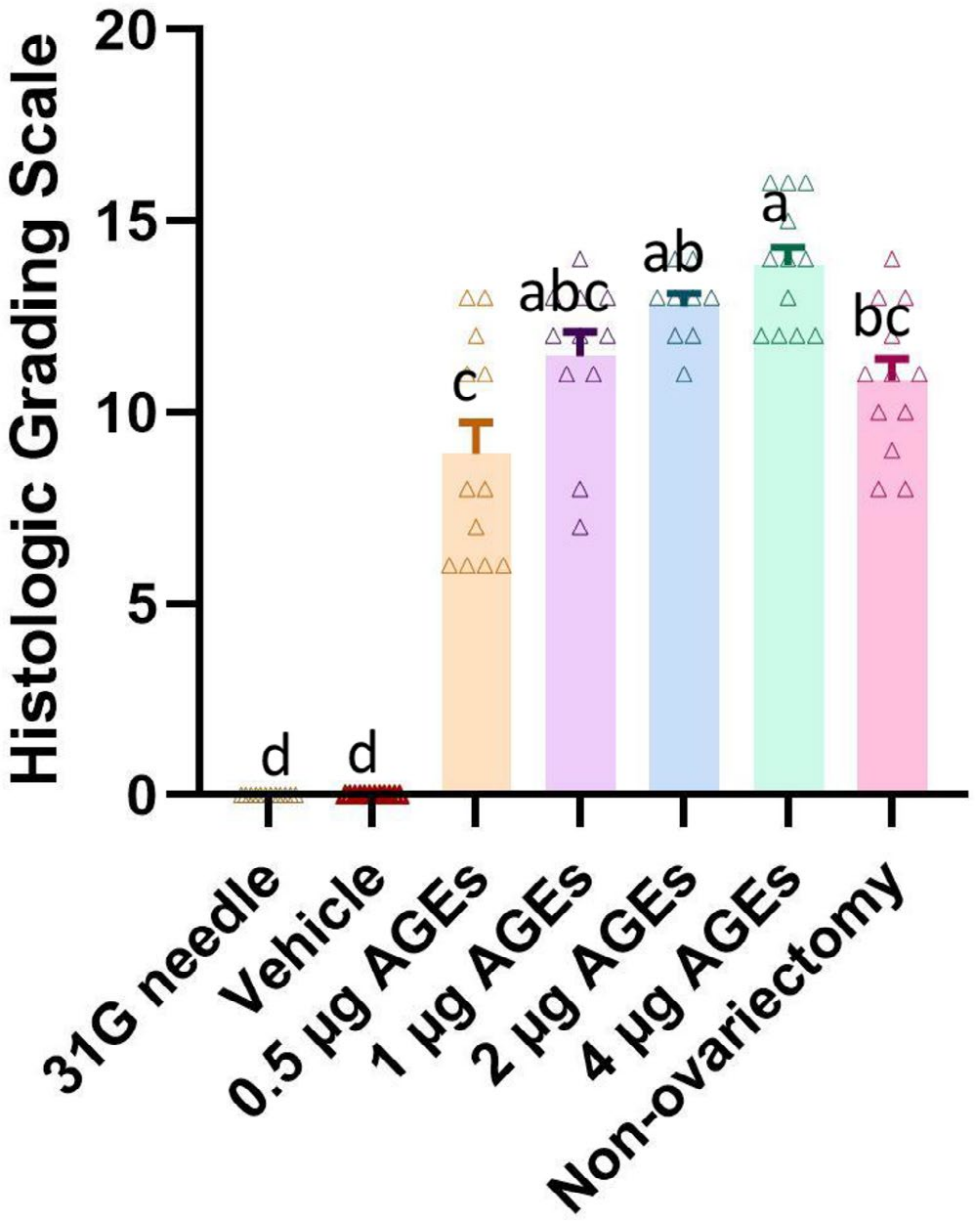
Statistical results of histological scores of rat coccygeal discs. Those labeled with the same letters indicate no statistical difference between groups, and those with different letters indicate a statistical difference between groups (*p* < 0.05).

Histological scores did not indicate a significant difference between the vehicle group and 31G needle group. The AGEs groups showed significantly higher scores compared to these controls, with higher AGEs doses correlating with higher scores (Figure 6) (Table S4).

### 3.5 Immunohistochemical Evaluation

By ANOVA and Tukey's multiple comparisons test, we obtained comparative results of Aggrecan and SOD2 in each group.

**FIGURE 7:**
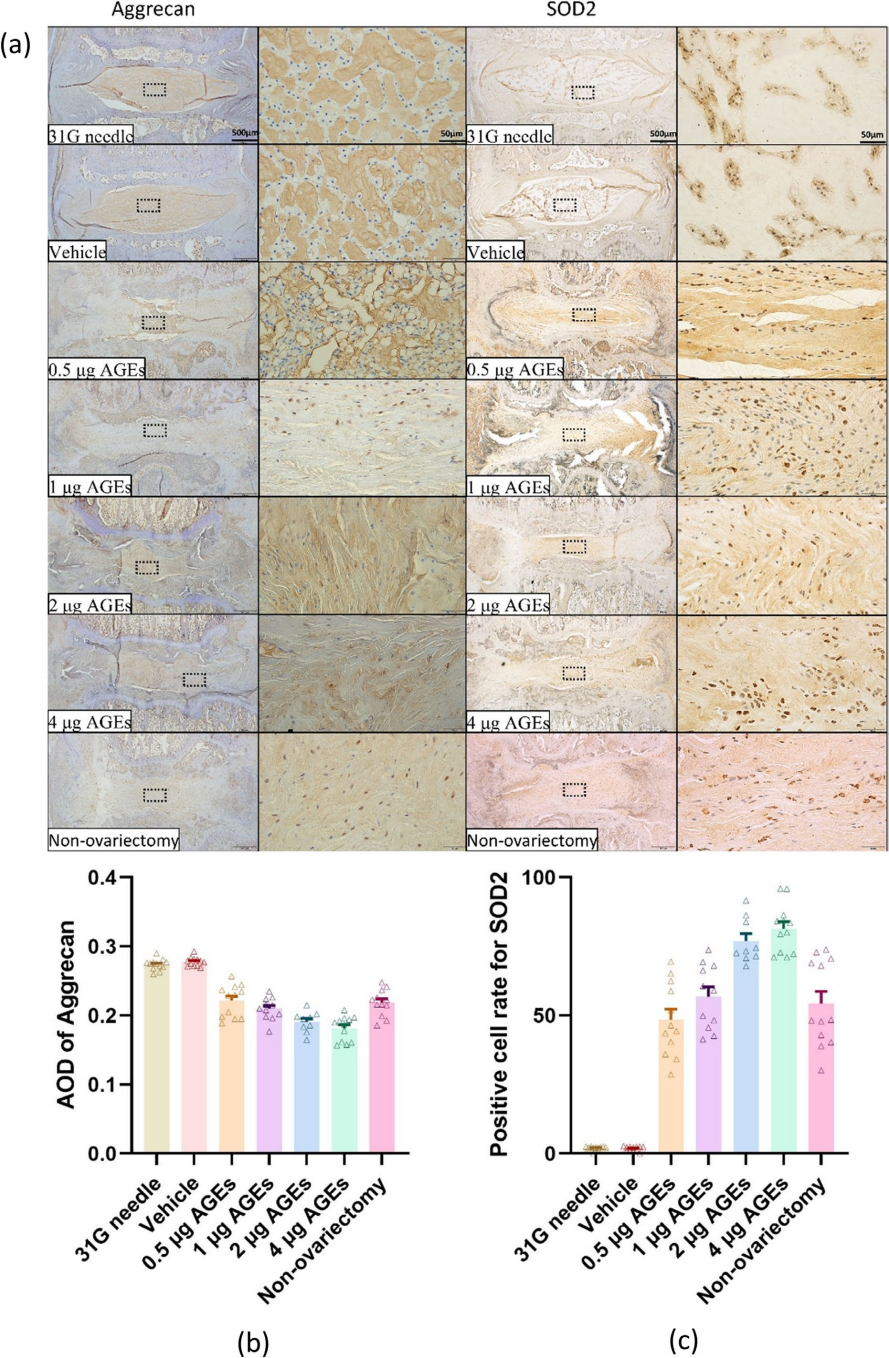
Immunohistochemical results of rat IVDs. (a) From left to right: Aggrecan expression in IVD, Aggrecan expression in nucleus pulposus, SOD2 expression in IVD, and SOD2 expression in nucleus pulposus. From top to bottom: 31G needle group, vehicle group, 0.5 μg AGEs group, 1 μg AGEs group, 2 μg AGEs group, 4 μg AGEs group, and non‐ovariectomy group. (b) Statistical results of Aggrecan expression in the nucleus pulposus of the coccygeal disc of rats. (c) Statistical results of SOD2 expression in the nucleus pulposus of the coccygeal disc of rats. Those labeled with the same letter indicate no statistical difference between groups, and those with different letters indicate a statistical difference between groups (*p* < 0.05).

IHC staining for Aggrecan revealed no significant difference between the 31G needle group and vehicle group. However, Aggrecan levels progressively decreased with higher doses of AGEs injection compared to the 31G needle group and vehicle group. No statistical differences were observed among the 0.5 μg AGEs group, 1 μg AGEs group, and non‐ovariectomy group, or between the 2 μg AGEs group and the 4 μg AGEs group. There was a significant reduction of Aggrecan level in the 2 μg AGEs group compared to the non‐ovariectomy group (Figure 7a,b) (Table S5).

For SOD2, IHC staining showed no significant difference between the 31G needle and vehicle groups. SOD2 levels increased progressively with higher AGEs doses. Compared with the 4 μg AGEs group, SOD2 levels were significantly lower in both the 0.5 μg AGEs group and the non‐ovariectomy group. Additionally, the SOD2 level was lower in the non‐ovariectomy group compared to the 2 μg AGEs group (Figure 7a,c) (Table S6).

## 4 Discussion

In this study, we established a simple, rapid, and high success rate (100% successful) animal model of IVDD by injecting AGEs into the coccygeal IVD of ovariectomized female rats. One of the characteristics of this model is that the degree of degeneration is adjustable based on the injection dose of AGEs. The modeling effect was verified by x‐ray, MRI, histological, and IHC evaluation. This provides an effective modeling method for future studies requiring disc models at various levels of degeneration.

Puncture needles used for drug injections can potentially induce IVDD, making needle size critical for successful modeling. Elmounedi et al. [32] investigated the effect of puncture needle size on IVDD in rats and found that the smaller the puncture needle size, the smaller the effect on IVD; they recommend 29G or finer puncture needles as ideal for drug injections. Issy et al. [33] found that 30G puncture needles combined with saline injections resulted in MRI signal changes in IVD. However, a noteworthy point is that their puncture needles stayed in the IVD for 5 min. For this study, we chose a 31G needle, which remained in the IVD for only 10 s post‐injection, minimizing potential damage. The results confirmed that the 31G puncture needle did not cause any IVDD signs on imaging or histology at 2 weeks postoperatively. In addition, 10‐s retention time was sufficient for the drug to diffuse within the IVD. This confirms that the 31G needle, coupled with 10 s retention time, is a reliable choice for drug delivery to rat IVDs.

AGEs have been used to induce IVDD. Liao et al. [23] and Song et al. [20] injected AGEs into rats' IVDs to establish IVDD models. AGEs were applied at 200 μg/mL (single dose: 2 μL), at 1‐week and 2‐week intervals. A total of 4 injections lead to 1.6 μg AGE. This injection method successfully induced degeneration of rat coccygeal IVDs. Jiang et al. [25] injected AGEs into the rat coccygeal IVD at 50 μg/mL, four times, 1 week apart. They injected 0.8 μg in total. Eventually, they also succeeded in inducing IVDD in the rats. Referring to these previous reports, the injection doses of AGEs used in our study were 0.5 to 4 μg. Compared to previous studies, we performed only one injection and induced IVDD within a much shorter period (2 weeks vs. 4–8 weeks). We may attribute this to the fact that AGEs promote NP cell death and IVDD progression by exacerbating oxidative stress, mitochondrial dysfunction, and inducing endoplasmic reticulum stress [20, 23, 34]. Furthermore, the induction of NP cell apoptosis by AGEs is dose‐ and time‐dependent. Studies have confirmed that increasing AGEs and prolonged stimulation lead to cytosolic and luminal Ca^2+^ changes in NP cells, causing endoplasmic reticulum stress and apoptosis [21, 35]. In these previous studies, the concentration of AGEs per injection was low (50 or 200 μg/mL), and the establishment of IVDD relied on the accumulation of AGEs caused by multiple injections. In contrast, the concentration of AGEs in our study reached a minimum of 500 μg/mL, and such a concentration of AGEs induced apoptosis and a decrease in ECM production of IVD cells in a much shorter period. Thus, we believe that performing only one injection of sufficient AGEs may be a better choice to establish an IVDD model. First, increasing the frequency of animal manipulations leads to anesthesia and excessive suffering of the rats, which increases the risk of death. Second, each puncture may damage cartilage endplates, with or without x‐rays, increasing risk with multiple injections. Third, compared to injections, a single AGE injection can significantly reduce study time while still inducing IVDD.

Previous studies have shown that AGEs contribute to IVDD by exacerbating oxidative stress and can affect the production of ECM in IVD [20, 21]. SOD2 is a protein that is involved in cellular defense against oxidative stress processes, whereas aggrecan is a major component of the ECM in IVD. We therefore chose these two proteins to verify the effect of AGEs on the oxidative stress process in IVD cells and their intervention in ECM synthesis.

In the current study, rats received 0.5, 1, 2, and 4 μg AGEs via a single injection into the IVDs. Mao et al. [36] found that 1 or 2 μL of PBS did not induce degeneration in rat IVDs. A 2.5 μL PBS injection decreased the radial height index and water content of the rat IVD significantly 2 weeks later. Thus, we controlled the injection volume to a maximum of 1 μL. Our results confirmed that 1 μL of PBS injected into the rat IVD did not induce IVDD, whereas, in the AGE injection groups, the degree of degeneration of the rat's IVD gradually increased with the increase of AGE injection. Our results also showed that the level of SOD2 was low in the healthy rat coccygeal IVD and that the IVDD was exacerbated by the increase in oxidative stress levels in the NP cells with increasing doses of AGE injections, accompanied by the increase of SOD2.

Estrogen receptors are expressed in IVD in humans and rats [10, 37]. Estrogen reduces apoptosis in rat NP cells under high glucose conditions by reducing reactive oxygen species, increasing ECM macromolecules [38] and inhibiting inflammatory factors such as IL‐1β and TNF‐α [39, 40]. In our study, the doses of AGEs injected into the coccygeal IVD of rats in the non‐ovariectomy group and the 2 μg AGEs group were the same; however, the non‐ovariectomy group showed lower expression of SOD2 and a lesser degree of degeneration. Estrogen secreted by rats slows IVDD progression. Ovariectomy is not necessary for AGE‐induced IVDD, but it may shorten the trial period. Researchers should decide whether to perform ovariectomy according to their experimental design.

Our study has some limitations. First, the current animal model was only applied to female rats and was not validated in male rats. Further testing is needed to check if the same dose causes the same degree of IVDD in male rats. Second, for animal welfare reasons, we did not validate all doses of AGEs in rats with preserved ovaries. However, our results show that ovary preservation prolongs IVDD models. Third, due to the complexity of human IVDD progression, the current animal model is unlikely to fully simulate human IVDD (a limitation of all animal models); however, it provides an important, insightful protocol for inducing IVDD via oxidative stress. In addition, the apoptosis‐related information and the mechanism relating to the protective effect of estrogen were not investigated in our study, and thus, we will continue to investigate the mechanism of IVDD caused by AGEs and the related signaling pathway in the future.

In summary, a single injection of AGEs to caudal disc can cause reliable and reproducible IVDD in ovariectomized female rats. Additionally, the endogenous estrogen might take a protective effect on the IVD to mitigate disc degeneration.

## Supporting information


**Table S1:** jsp270114‐sup‐0001‐TableS1‐S6.docx. *p*‐value for comparison of Disc Height Index among different subgroups. (*p < 0.05, **p < 0.01).
**Table S2:** *p*‐value for comparison of Pfrrmann's classification among different subgroups. (*p < 0.05, **p < 0.01).
**Table S3:** *p*‐value for comparison of MRI index among different subgroups. (*p < 0.05, **p < 0.01).
**Table S4:** *p*‐value for comparison of histologic grading scale among different subgroups. (*p < 0.05, **p < 0.01).
**Table S5:** *p*‐value for comparison of AOD of aggrecan among different subgroups. (*p < 0.05, **p < 0.01).
**Table S6:** *p*‐value for comparison of positive cell rate for SOD2 among different subgroups. (*p < 0.05, **p < 0.01).
