# Supplementary material for: Advanced Glycation End Products Induce Caudal Disc Degeneration in Ovariectomized Female Rats
Source: JOR Spine. 2025 Sep 9;8(3):e70114. doi: 10.1002/jsp2.70114 (PMC12419273; doi:10.1002/jsp2.70114)
Supplement: Supplementary file 1 — Table S1: jsp270114‐sup‐0001‐TableS1‐S6.docx. p‐value for comparison of Disc Height Index among different subgroups. (*p < 0.05, **p < 0.01). Table S2: p‐value for comparison of Pfrrmann's classification among different subgroups. (*p < 0.05, **p < 0.01). Table S3: p‐value for comparison of MRI index among different subgroups. (*p < 0.05, **p < 0.01). Table S4: p‐value for comparison of histologic grading scale among different subgroups. (*p < 0.05, **p < 0.01). Table S5: p‐value for comparison of AOD of aggrecan among different subgroups. (*p < 0.05, **p < 0.01). Table S6: p‐value for comparison of positive cell rate for SOD2 among different subgroups. (*p < 0.05, **p < 0.01). [file JSP2-8-e70114-s001.docx]

Table S1 P value for comparison of Disc Height Index among different subgroups. (*p<0.05, **p<0.01)

| Group | 31G needle | Vehicle | 0.5μg AGEs | 1μg AGEs | 2μg AGEs | 4μg AGEs |
| --- | --- | --- | --- | --- | --- | --- |
| Vehicle | 1.000 |  |  |  |  |  |
| 0.5μg AGEs | 0.000** | 0.000** |  |  |  |  |
| 1μg AGEs | 0.000** | 0.000** | 1.000 |  |  |  |
| 2μg AGEs | 0.000** | 0.000** | 0.020* | 0.025* |  |  |
| 4μg AGEs | 0.000** | 0.000** | 0.000** | 0.000** | 0.215 |  |
| non-ovariectomy | 0.000** | 0.000** | 1.000 | 1.000 | 0.009* | 0.000** |

Table S2 P value for comparison of Pfrrmann's classification among different subgroups. (*p<0.05, **p<0.01)

| Group | 31G needle | Vehicle | 0.5μg AGEs | 1μg AGEs | 2μg AGEs | 4μg AGEs |
| --- | --- | --- | --- | --- | --- | --- |
| Vehicle | 1.000 |  |  |  |  |  |
| 0.5μg AGEs | 0.000** | 0.000** |  |  |  |  |
| 1μg AGEs | 0.000** | 0.000** | 0.731 |  |  |  |
| 2μg AGEs | 0.000** | 0.000** | 0.067 | 0.139 |  |  |
| 4μg AGEs | 0.000** | 0.000** | 0.020* | 0.053 | 0.748 |  |
| non-ovariectomy | 0.001** | 0.001** | 0.862 | 0.608 | 0.046* | 0.012* |

Table S3 P value for comparison of MRI index among different subgroups. (*p<0.05, **p<0.01)

| Group | 31G needle | Vehicle | 0.5μg AGEs | 1μg AGEs | 2μg AGEs | 4μg AGEs |
| --- | --- | --- | --- | --- | --- | --- |
| Vehicle | 0.819 |  |  |  |  |  |
| 0.5μg AGEs | 0.000** | 0.001** |  |  |  |  |
| 1μg AGEs | 0.001** | 0.001** | 0.957 |  |  |  |
| 2μg AGEs | 0.000** | 0.000** | 0.159 | 0.153 |  |  |
| 4μg AGEs | 0.000** | 0.000** | 0.006** | 0.006** | 0.255 |  |
| non-ovariectomy | 0.001** | 0.002** | 0.785 | 0.832 | 0.097 | 0.003** |

Table S4 P value for comparison of histologic grading scale among different subgroups. (*p<0.05, **p<0.01)

| Group | 31G needle | Vehicle | 0.5μg AGEs | 1μg AGEs | 2μg AGEs | 4μg AGEs |
| --- | --- | --- | --- | --- | --- | --- |
| Vehicle | 1.000 |  |  |  |  |  |
| 0.5μg AGEs | 0.005** | 0.005** |  |  |  |  |
| 1μg AGEs | 0.000** | 0.000** | 0.181 |  |  |  |
| 2μg AGEs | 0.000** | 0.000** | 0.029* | 0.371 |  |  |
| 4μg AGEs | 0.000** | 0.000** | 0.002** | 0.104 | 0.530 |  |
| non-ovariectomy | 0.000** | 0.000** | 0.416 | 0.587 | 0.154 | 0.027* |

Table S5 P value for comparison of AOD of aggrecan among different subgroups. (*p<0.05, **p<0.01)

| Group | 31G needle | Vehicle | 0.5μg AGEs | 1μg AGEs | 2μg AGEs | 4μg AGEs |
| --- | --- | --- | --- | --- | --- | --- |
| Vehicle | 0.996 |  |  |  |  |  |
| 0.5μg AGEs | 0.000** | 0.000** |  |  |  |  |
| 1μg AGEs | 0.000** | 0.000** | 0.599 |  |  |  |
| 2μg AGEs | 0.000** | 0.000** | 0.001** | 0.145 |  |  |
| 4μg AGEs | 0.000** | 0.000** | 0.000** | 0.002** | 0.854 |  |
| non-ovariectomy | 0.000** | 0.000** | 1.000 | 0.839 | 0.004** | 0.000** |

Table S6 P value for comparison of positive cell rate for SOD2 among different subgroups. (*p<0.05, **p<0.01)

| Group | 31G needle | Vehicle | 0.5μg AGEs | 1μg AGEs | 2μg AGEs | 4μg AGEs |
| --- | --- | --- | --- | --- | --- | --- |
| Vehicle | 1.000 |  |  |  |  |  |
| 0.5μg AGEs | 0.000** | 0.000** |  |  |  |  |
| 1μg AGEs | 0.000** | 0.000** | 0.425 |  |  |  |
| 2μg AGEs | 0.000** | 0.000** | 0.000** | 0.001** |  |  |
| 4μg AGEs | 0.000** | 0.000** | 0.000** | 0.000** | 0.949 |  |
| non-ovariectomy | 0.000** | 0.000** | 0.791 | 0.996 | 0.000** | 0.000** |
